# Supplementary material for: Circulating HPV cDNA in the blood as a reliable biomarker for cervical cancer: A meta-analysis
Source: PLoS One. 2020 Feb 6;15(2):e0224001. doi: 10.1371/journal.pone.0224001 (PMC7004305; doi:10.1371/journal.pone.0224001)
Supplement: S2 Table — (DOCX) [file pone.0224001.s002.docx]

S2 Table. The primers of the detected HPV cDNA in 10 enrolled studies

| studies | HPV cDNA primers | Probes | method |
| --- | --- | --- | --- |
| Pornthanakasem W[10] | HPV E6 gene  Up:5’ CGGTTSAACCGAAAMCGG3’  Down:5’ CGGTCGGGACCGAAAACGG3’ | Not provided in the literature | qPCR |
| Dong SM[25] | HPV E7 gene  UP: 5’ ATTAAATGACAGCTCAGAGGA3’  Down:5’GCTTTGTACGCACAACCGAAGC3’ | 5’-(FAM)CCCCAAAGGACTCAAAGAACCT-(TAMRA)-3_ | qPCR |
| Hsu KF[30] | HPV E7 gene(the same as above)  UP: 5’ ATTAAATGACAGCTCAGAGGA3’  Down:5’GCTTTGTACGCACAACCGAAGC3’ | Not provided in the literature | qPCR |
| Sathish N[31] | HPV E7 gene(the same as above)  MY09/MY11:  UP: 5’ ATTAAATGACAGCTCAGAGGA3’  Down:5’GCTTTGTACGCACAACCGAAGC3’ | Not provided in the literature | PCR+RFLP |
| Yang HJ[26] | HPV 16  UP:5’ ATCATCAAGAACACGTAGAG3’  Down:5’ GATCAG TTGTCTCTGGTTGCA AAT3’  HPV 18  Up:5’ GATTTCACAACATAGCTGGG3’  Down:5’TGCCTTAGGTCCATGCATAC3’ | probe: 5’-FAM-CGACTC CAA CGA CGC AGA GA-TAMRA-3’ | qPCR |
| Wei YC[32] | HPV L1 gene  The first primers:MY11/MY09  The resulted primers:  Up:5’ TTTGYTGGGGTAATCARYTRT3’  Down:5’ TAARTCWSYAGAAAACTTTTC3’(W=A+T, Y=C+T, M=A+C,S=C+G and R=A+G) | Not provided in the literature | Nested  qPCR |
| Jaberipour M[33] | HPV E6 gene  Commercial kits for quantification of human papilloma virus genomes(Advanced kit version, PrimerDesign, Southampton, UK) with specific primers which was not showed in the article. | Not provided in the literature | qPCR |
| Campitelli M[24] | HPV E7 gene  The primers used in this article was referenced to the study < Frequent genomic structural alterations at HPV insertion sites in cervical carcinoma> . | The probes used in this article was also referenced to the study < Frequent genomic structural alterations at HPV insertion sites in cervical carcinoma> . | DIPS-PCR |
| Jeannot E[27] | HPV 16 E7 gene  Up:5’ TCCAGCTGGACAAGCAGAAC3’  Down:5’ CACAACCGAAGCGTAGAGTC3’ | 5’-FAM-AACCACAACGTCACACAA-  30 | ddPCR |
| Kang Z[17] | HPV 16 E7 gene  Up :5’AGGATGAAATAGATGGTCCA3’  Down:5’GTAGAGTCACACTTGCAACA3’  HPV 18 E7 gene  Up:5’TGAAGCCAGAATTGAGCTAGT3’  Down:5’CAGAAACAGCTGCTGGAATG3’ | HPV 16  5’-FAM-TGGACAAGCAGAACCGGACAGAGCCC3’  HPV 18  5’-FAM-AGTAGAAAGCTCAGCAGACGACCTTCGAG-3’ | ddPCR |
