# Supplementary material for: Circulating HPV cDNA in the blood as a reliable biomarker for cervical cancer: A meta-analysis
Source: PLoS One. 2020 Feb 6;15(2):e0224001. doi: 10.1371/journal.pone.0224001 (PMC7004305; doi:10.1371/journal.pone.0224001)

search strategy:

No limitations were set with regard to the start date for publication, and the search ended on March 18, 2019. The following search terms were used: “cervical cancer AND HPV cDNA”, “cervix cancer AND ctDNA”, “cervical carcinoma AND ctDNA” OR “circulating DNA AND cervical cancer”. Various alterations in spelling and abbreviations were also used as search terms. Titles and abstracts were carefully screened for relevance, and duplicates were removed. The full text of each report that met the preliminary criteria was retrieved and assessed for inclusion into this meta-analysis.

Search terms: ((((cervical cancer AND HPV cDNA)) OR ((cervix cancer) AND ctDNA)) OR ((cervical carcinoma) AND ctDNA)) OR ((circulating DNA) AND cervical cancer)

Search Results:

Totally 237 items:


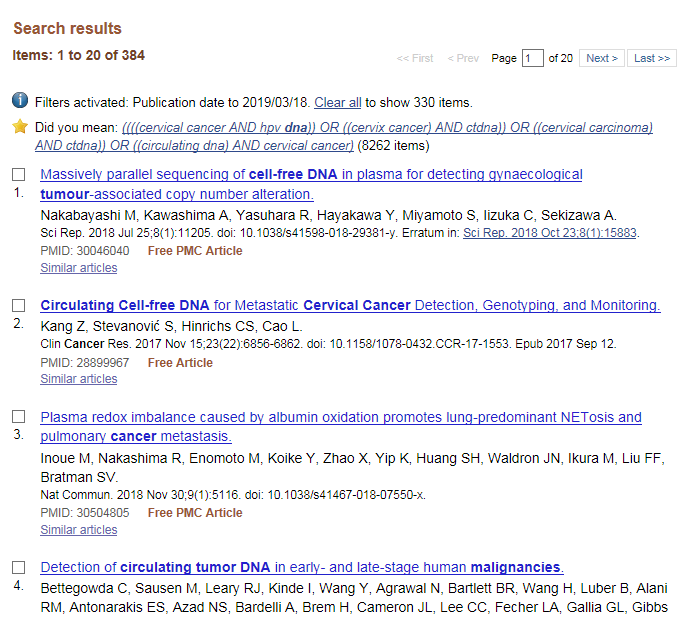

Supplement: S1 Fig — (DOCX) [file pone.0224001.s004.docx]
