# Supplementary material for: Circulating HPV cDNA in the blood as a reliable biomarker for cervical cancer: A meta-analysis
Source: PLoS One. 2020 Feb 6;15(2):e0224001. doi: 10.1371/journal.pone.0224001 (PMC7004305; doi:10.1371/journal.pone.0224001)
Supplement: Attachment — Submitted filename: a_rebuttal_letter.docx [file pone.0224001.s005.docx]

[2019-11-07]

Manuscript number: PONE-D-19-27050

Circulating HPV cDNA in the blood as a reliable biomarker for cervical cancer: a meta-analysis.

Dear Academic Editor Peter van Dam:

Thank you for your time and effort in reviewing our manuscript and providing us with comprehensive and valuable feedback.

Please find below the clarifications for the concerns raised:

**Comment:** Before sending this paper to the reviewers I feel the authors should rewrite the present paper. It contains valuable information but I feel it is non-sense to perform a meta-analysis combining HPV and non-HPV tests. The meta-analysis should mainly focus on HPV cDNA. The studies looking at non-HPV DNA (Table 1: studies 5, 11, 14, and 15) should be analyzed separately. For the HPV cDNA the probes used in the different studies should be mentioned in a Table.

**Clarifications:** We appreciate your suggestion on focusing on the data from HPV cDNA in our meta-analysis. Based on the findings from our original manuscript, we believe that, after integration into the human chromosome, HPV cDNA behaves like other functional genes. Subgroup analysis also showed that there were no significant differences between HPV cDNA and ctDNA of other genes. However, too many miscellaneous ctDNAs may potentially result in heterogeneity that is incompatible with the setup for meta-analysis. Therefore, we have excluded studies on non-HPV cDNA and solely focused on studies including HPV cDNA. The tables and figures have also been replaced in the revised manuscript.

We have ensured conformance to all the journal guidelines in our revised manuscript.

1. We have revised our manuscript as per the style guidelines prescribed by PLoS One.

2. Supporting Information files have been provided at the end of the revised manuscript.

3. The discussion has been rewritten and the text has been citated with the relevant publications.

4. The full search terms to be used in the PubMed database has been provided in the Supplementary Information.

5. We have included information about the financial support lent to two of our authors (Tingwang Jiang and Zhixiang Zhuang) from the respective funding agencies. The funders had no role in designing the study, data collection and analysis, decision to publish, or preparation of the manuscript.

The revised parts of the manuscript have been highlighted in yellow. Thank you for your consideration. I look forward to your response.

Sincerely,

Dr. Chuandan Wan

Changshu Medical Examination Institute

No. 36 Qingduntang Road, Changshu City, Jiangsu Province, Changshu, 215500, China

Phone number:18962480440

Telephone number:0512-52901591

E-mail: cd_wan@163.com
