# Supplementary material for: Circulating HPV cDNA in the blood as a reliable biomarker for cervical cancer: A meta-analysis
Source: PLoS One. 2020 Feb 6;15(2):e0224001. doi: 10.1371/journal.pone.0224001 (PMC7004305; doi:10.1371/journal.pone.0224001)
Supplement: Attachment — Submitted filename: a_rebuttal_letter.docx [file pone.0224001.s006.docx]

[2019-11-07]

Manuscript number: PONE-D-19-27050R1

Circulating HPV cDNA in the blood as a reliable biomarker for cervical cancer: a meta-analysis.

Dear Academic Editor and reviewers:

Thank you for your time and effort in reviewing our manuscript and providing us with comprehensive and valuable feedback.

Please find below the clarifications for the concerns raised:

**Review Comment:** For Table 1, please add the number of cases, non-cases, TP, FP, FN, TN and cut-off values (if any) for each study. The sensitivities of No. 1, 2 and 4 studies are different from those reported in Table S1.

**Clarifications:** The number of TP, FP, FN, TN for each study has been added in Table1. Meanwhile some unimportant data have been deleted from Table 1. The sensitivities of No.1, 2 and 4 studies in Table S1 also have been corrected.

**Review Comment:** For quality assessment, please provide a table to report the QUADAS assessment scores for each included study individually.

**Clarifications:** QUASAS assessment scores were added to Table 1.

**Review Comment:** Page 10, under Quality assessment, the 2nd line “The included 15 studies…” should be “The included 10 studies...”

**Clarifications:** In the last revision, the original 15 papers were included in this meta-analysis. According to the requirements, the theme was re-locked on HPV cDNA. The original 15 articles were reduced to 10. We forgot to revise them here. Now it has been corrected.

**Review Comment:** In Figure 3, it is not clear if random effects model was used for pooled sensitivity or specificity. In Figure 3, please add meta-analysis analysis for DOR.

**Clarifications:** In Figure 3, both sensitivity and specificity analysis are obtained under the random effects mode. DOR results have also been added to Table 3.

**Review Comment:** In subgroup analysis, race is a factor, but in Table 2A, no race is included. The term of race or region and their contents should be consistent through the text and tables.

**Clarifications:** The term of race or region and their contents have been checked consistently through the text and tables.

**Review Comment:** What are the estimates in Figure 5? For sensitivity analysis please include pooled results for each of major outcomes (sensitivity, specificity, and DOR).

**Clarifications:** Sensitivity, specificity, and DOR aren’t generally provided in sensitivity analysis of meta review literatures (including sensitivity analysis of meta literature in PLoS One and other journals). The sensitivity analysis here is to examine the effect of a single study on the total combined effect (estimates and their 95%CI). Figure 5 showed that the results were relatively stable and reliable.

**Review Comment:** Page 18, in Discussion, the statement, “Most studies employed a qPCR method that demonstrated relatively high sensitivity and specificity”, does not reflect the truth. From Tables 1 and S1 (even though the numbers are not consistent between these two tables), those studies with qPCR method show very low sensitivities.

**Clarifications:** The original sentence has been revised to: “Most studies employed a qPCR method that demonstrated relatively high specificity but low sensitivity. The sensitivity of qPCR is difficult to detect very small amounts of circulating nucleic acids in blood.” It is true that MSP-PCR and ddPCR were more accurate for detecting HPV cDNA in patients than qPCR. The reasons for these results are also discussed in text.

**Review Comment:** From Table 1, it seems DIPS-PCR and ddPCR would give high sensitivity but not other method. But in Discussion, authors don’t think the test methods would contribute heterogeneity, which is not convincing.

**Clarifications:** From the perspective of raw data, differences in methodology can indeed bring about greater heterogeneity, but meta-analysis shows that the test methods do not contribute statistically significant heterogeneity. In discussion, we suggested some possible causes of heterogeneity. Of cause, further studies with large sample sizes and more details, e.g., race, specimen features, and tumor properties, are needed to confirm these suggestions.

The revised parts of the manuscript have been highlighted in yellow. Thank you for your consideration. I look forward to your response.

Sincerely,

Dr. Chuandan Wan

Changshu Medical Examination Institute

No. 36 Qingduntang Road, Changshu City, Jiangsu Province, Changshu, 215500, China

Phone number:18962480440

Telephone number:0512-52901591

E-mail: cd_wan@163.com
